# 0597. The relation between intestinal intramucosal ph and stress hormones in pig hemorrhagic shock model

**DOI:** 10.1186/2197-425X-2-S1-P40

**Published:** 2014-09-26

**Authors:** M Arai, S Ito, Y Kosaka, M Toda, M Kuroiwa, H Okamoto

**Affiliations:** Kitasato University, Anesthesia and Intensive Care Medicine, Sagamihara, Japan; Kitasato University, School of Medicine, Anesthesiology, Sagamihara, Japan

## Introduction

It is known that intestinal intramucosal pH (pHi) is a good parameter of tissue perfusion in critical patients. However, physiological meaning of pHi value related to other physiological parameter, such as stress hormone, is not well known. The purpose of the study is to clarify the importance of monitoring pHi during hemorrhagic shock in pig model. The relation between pHi, oxygen supply and demand balance at intestinal region and stress hormones are discussed.

## Methods

The studies were performed on 6 pigs (body weight 25±4 kg). Anesthesia was induced by inhalation of isoflurane 3-5% and after endotracheal intubation, pigs were placed on a positive pressure ventilation. Anesthesia was maintained with pancronium and isoflurane 2%. A catheter was inserted through carotid artery to measure aortic blood pressure and to sample blood. Ringer's Lactated solution was infused throughout the experiment at 10 ml/kg/hr. The pig's abdomen was opened through a midline incision and the electromagnetic flow probe was placed on the root of superior mesenteric artery (SMA). Tonomitor® (Tonometrics) was inserted from antimesentric region of jejunum. A polyvinyl tube was inserted to superior mesenteric vein (SMV) to sample blood.

Protocol: Control data were obtained after 30 min stabilization period (Baseline; S1). Then the pigs were bled gradually and MAP was maintained at 40mmHg for 1 hours (Shock Phase; S2). Pigs were resuscitated with whole blood (Resuscitation Phase 1; S3). Additional 1 hour was evaluated for post-resuscitation phase (S4).

Measurements: Jejunal pHi was calculated by the luminal PCO2, obtained with a balloon tonometer, and arterial bicarbonate concentration. Arterial blood samples were taken for analysis of adrenaline (ADR), noradrenaline (NA), angiotensin II (Ang II) and arterial blood gas. SMV blood samples were taken for analysis of lactate/pylvate acid (sma L/P), and blood gas (PsmvCO2).

## Results

**Figure 1:**
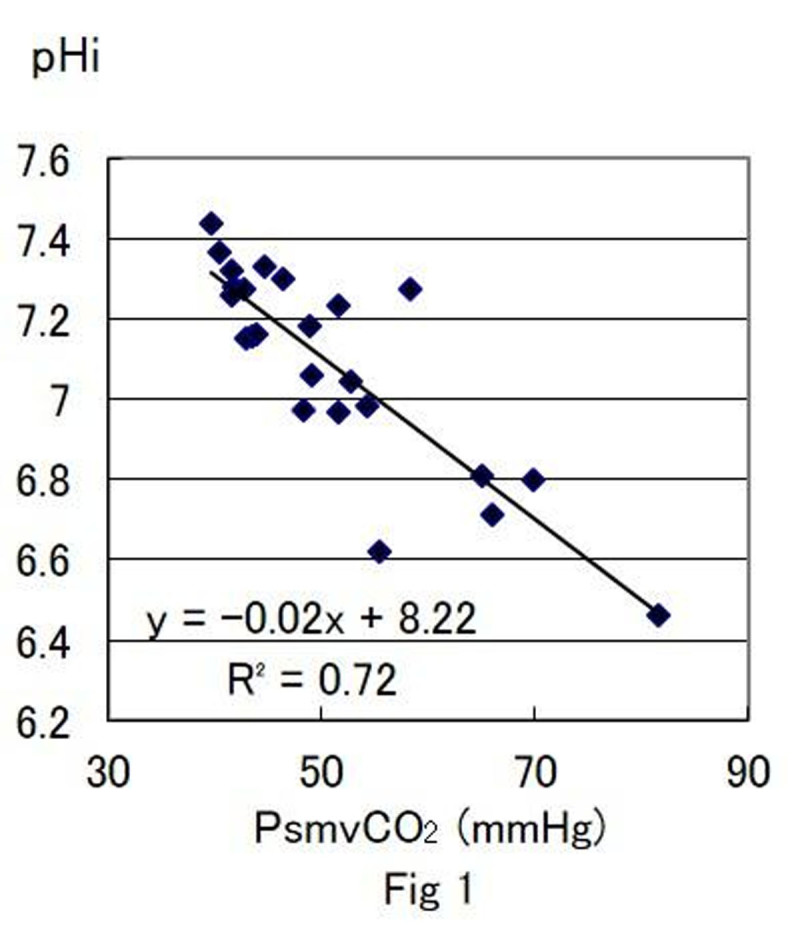
Figure 1

**Figure 2:**
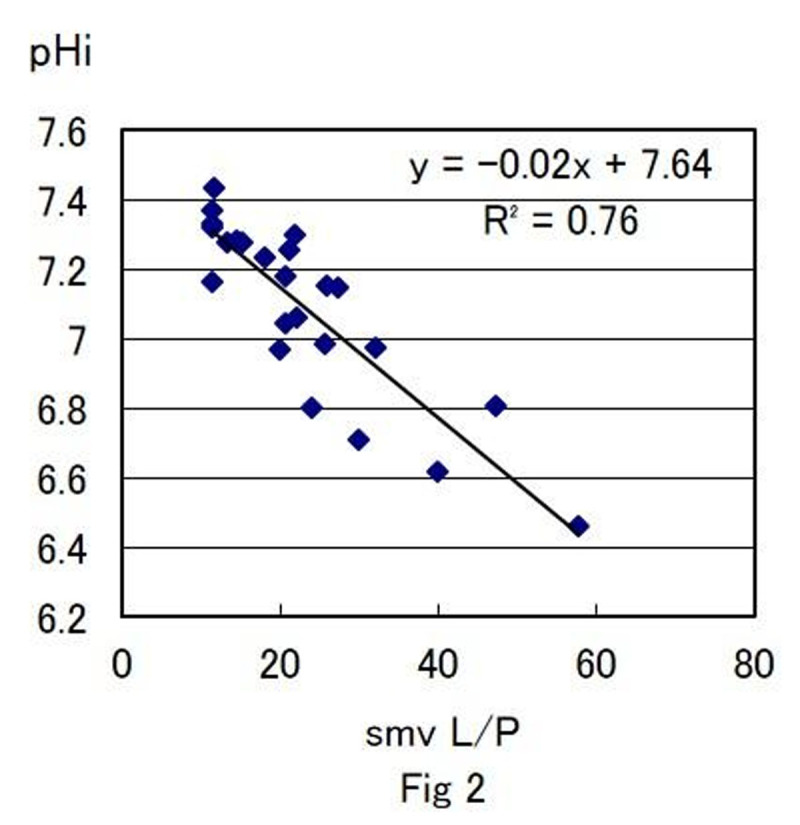
Figure 2

At shock phase, SMA flow decreased to 40% from the baseline (p< 0.01) and pHi decreased from 7.4±0.2 to 6.8±0.2 (p< 0.01). The correlation between pHi and other parameters are as follows, pHi vs PsmvCO2; y=-0.02x+8.22 (r2=0.72) (Fig 1), pHi vs smv L/P ; y=-0.02x+7.64 (r2=0.76) (Fig 2), pHi vs ADR (pg/ml); y=-0.09ln(x)+7.74 (r2=0.72), pHi vs NA (pg/ml); y=-0.09ln(x)+7.79 (r2=0.74), pHi vs Ang II (pg/ml); y=-0.11ln(x)+7.71 (r2=0.75).

## Discussion

Changes of pHi value during hemorrhagic shock was correlated with PsmvCO2, smv L/P and stress hormones (ADR, NA, Ang II). It is considered that change in pHi show the anaerobic metabolism status of the intestinal tissue which indicate tissue hypoxia of the intestine induced by hemorrhagic shock and increase in sympathetic nerve activity. pHi was strongly correlated to other physiological parameters which indicate that pHi is significance parameter of hemorrhagic shock.

## Conclusions

pHi is significant parameter of tissue hypoperfusion and sympathetic nerve activity during hemorrhagic shock.

